# Regional Language Speech Recognition from Bone-Conducted Speech Signals through Different Deep Learning Architectures

**DOI:** 10.1155/2022/4473952

**Published:** 2022-08-25

**Authors:** Venkata Subbaiah Putta, A. Selwin Mich Priyadharson, Venkatesa Prabhu Sundramurthy

**Affiliations:** ^1^Department of Electronics and Communication Engineering, Vel Tech Rangarajan Dr. Sagunthala R&D Institute of Science and Technology, Avadi, Chennai, India; ^2^Center of Excellence for Bioprocess and Biotechnology, Department of Chemical Engineering, College of Biological and Chemical Engineering, Addis Ababa Science and Technology University, Addis Ababa, Ethiopia

## Abstract

Bone-conducted microphone (BCM) senses vibrations from bones in the skull during speech to electrical audio signal. When transmitting speech signals, bone-conduction microphones (BCMs) capture speech signals based on the vibrations of the speaker's skull and have better noise-resistance capabilities than standard air-conduction microphones (ACMs). BCMs have a different frequency response than ACMs because they only capture the low-frequency portion of speech signals. When we replace an ACM with a BCM, we may get satisfactory noise suppression results, but the speech quality and intelligibility may suffer due to the nature of the solid vibration. Mismatched BCM and ACM characteristics can also have an impact on ASR performance, and it is impossible to recreate a new ASR system using voice data from BCMs. The speech intelligibility of a BCM-conducted speech signal is determined by the location of the bone used to acquire the signal and accurately model phonemes of words. Deep learning techniques such as neural network have traditionally been used for speech recognition. However, neural networks have a high computational cost and are unable to model phonemes in signals. In this paper, the intelligibility of BCM signal speech was evaluated for different bone locations, namely the right ramus, larynx, and right mastoid. Listener and deep learning architectures such as CapsuleNet, UNet, and S-Net were used to acquire the BCM signal for Tamil words and evaluate speech intelligibility. As validated by the listener and deep learning architectures, the Larynx bone location improves speech intelligibility.

## 1. Introduction

The speech quality and intelligibility degrade due to ambient noise and implant location of air-conducted and bone-conducted devices. The speech intelligibility of noise-affected speech improves by noise suppression techniques. background noise, including musical noise, babbling noise, coloured noise, and nonstationary noise. In a speech recognition system, the noise suppresses automatically by filters such as Wiener and Kalman filter, noise subtraction techniques, and speech enhancement algorithm. However, the residual noise signal is caused by the nonlinear nature of the noise signal. The residual noise seriously affects speech intelligibility and recognition. Traditionally, noise suppression from speech signals has been accomplished by estimating the power spectrum of the noise signal. Because noise signals are nonlinear, power spectrum estimation is inaccurate. Due to the presence of residual noise, the obtained speech signal has reduced speech intelligibility and perception. Deep learning methods improve speech intelligibility and perception by suppressing nonlinear noise signals. The early fusion and late fusion of ensemble learning strategy along with convolutional neural network enhance speech signal obtained with bone-conducted microphone (BCM). The acoustic characteristics of BCM and air conducted microphone (ACM) signal learned by ensemble approach and convolutional neural network for speech signal enhancement [1].

The BCM and ACM conducted speech signal obtained in the noisy environment of 61.7 dBA to 73.9 dBA, transform to match with each other by deep denoising autoencoder. The speech recognition accuracy improves by adjusting the weight of the speech intelligibility index [2]. The MED-EL bonebridge device speech perception was evaluated with a tone audiogram. The MED-EL bonebridge device has improved speech perception upon implantation. The implanted device's speech perception was tested with Freiburg monosyllable [3].

Speech perception is enabled with a transcutaneous bone-conduction implant (BCI BB) placed near to the mastoid bone's sinodural angle. The speech perception of the device evaluates with functional hearing gain. The BCI BB provides better speech perception under a noisy environment [4].

The Radioear B-71 bone vibrator and TDH-39 earphoneme speech perception were evaluated under quiet, pink, white, and babble conditions with Callsign Acquisition Test. The device's speech intelligibility was tested for mastoid and condyle locations. The speech intelligibility varied for gender due to background noise validated by post hoc analysis [5].

The speech intelligibility of bone-conducted ultrasound (BCU) and air-conducted ultrasound (ACU) signal of ceramic vibrator placed at mastoid region was evaluated with ANOVA test. The ACU speech intelligibility increased with a higher sound level compared to BCU [6].

Performance evaluation of the B-72 device is with regard to background noise, voice gender, and ear position. The modified rhyme test (MRT) was conducted with Fonix FA-12 audiometer and Telephonics TDH-39P earphoneme. The MRT results show condyle region increase speech intelligibility compared to the mastoid region [7].

## 2. Related Works

Table 1 explains the characteristics of the existing models.

Using noise-resistant recording devices is a simple way to collect less distorted speech signals. As previously stated, a BCM records signals via bone vibrations and is thus less sensitive to air background noise than an ACM. However, BCM-recorded speech signals frequently suffer from a loss of high acoustic-frequency components, which was addressed and partially alleviated by the BCM-to-ACM conversion technique.

## 3. Methodology

**Figure 1:**
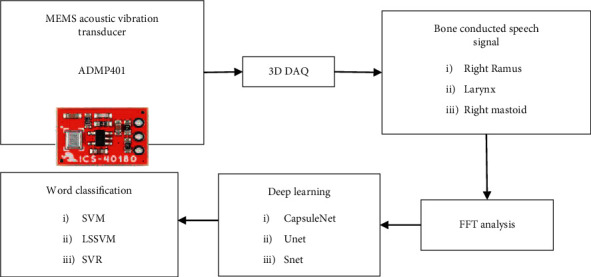
Overview of speech signal processing.

The study of speech signals and signal processing methods is known as speech processing. Because the signals are typically processed in digital form, speech processing can be thought of as a subset of digital signal processing applied to speech signals. The BCM speech acquires with MEMS acoustic sensor. The transducer converts vibrations induced at the bones of the skull to a spectral-rich electrical signal. The bones conduct vibrations from the vocal tract during speech. The vocal track causes vibrations on bones such as right ramus, larynx, and right mastoid as shown in Figure 1.

**Figure 2:**
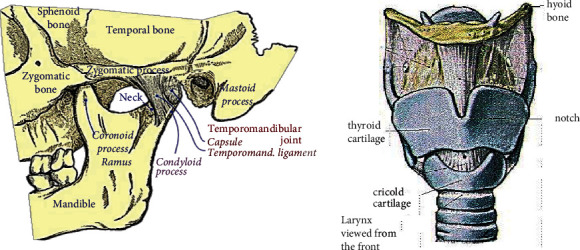
Bone location in skull and throat.

The speech stimuli involved in the study were five common words from the Tamil langue as shown in Table 2. The words are frequently used in conversation and represent Tamil language phonetic characteristics. The Tamil words spoken by male at 60 dB were recorded in a quiet environment with microphone placed at three feet from lips sampled at 22 kHz. Similarly, the words were recorded with an ADMP401 microphone placed at the right ramus, larynx, and right mastoid as shown in Figure 2. The ADMP401 was positioned over bone and prevents from drifting during speech with a headband. The ADMP401 signal was amplified by a class B power amplifier and recorded with Hp laptop and Sigview software.

### 3.1. CapsuleNet

A capsule network trained to detect objects in this database improved model accuracy by 45 percent when compared to traditional CNN models.

### 3.2. UNet

A general convolutional neural network focuses on image classification, where the input is an image and the output is one label, but in biomedical cases, we must not only determine whether disease exists but also localise the area of abnormality. UNet is committed to resolving this issue. It can localise and distinguish borders by performing classification on every pixel, so the input and output are the same sizes.

### 3.3. S-Net

S-Net was the first parallel neural network implementation. It employs the data division method, and the system employs one server and any number of clients. It was written in the C programming language. TCP/IP sockets are used by clients to connect to the server. Each client receives their own thread. Each client computes update matrices for his portion of the data (bunch-size/N), sends them to the server, and then waits for a response. When the server is aware of all update matrices, the main thread updates the weight. When the update is complete, the server sends new weights to clients via threaded client communication.

When compared to other methods, CapsuleNet, UNet, and S-Net recognised Tamil words accurately for BCM signals obtained from the larynx bone.

## 4. Results and Discussion

**Figure 3:**
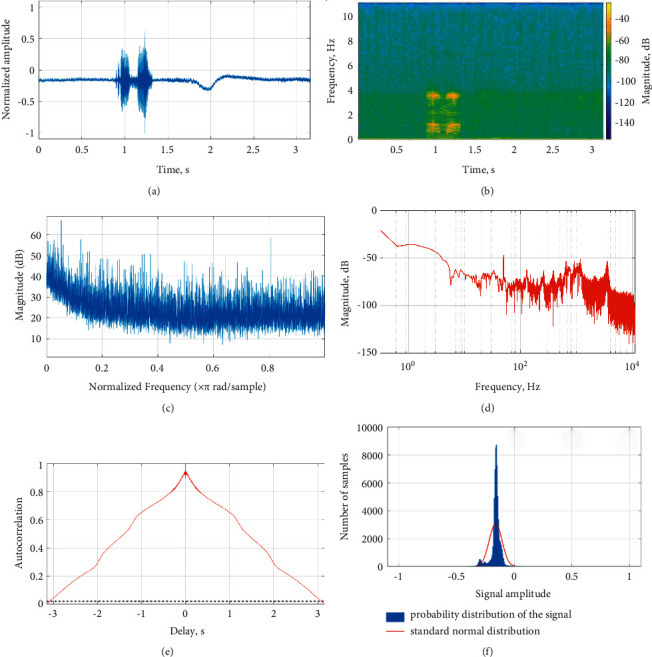
“Amam” speech signal waveform, spectrogram, magnitude response, amplitude spectrum, autocorrelation, and probability distribution. (a) “Amam” speech signal. (b) “Amam” signal spectrogram. (c) “Amam” signal magnitude response. (d) “Amam” signal amplitude spectrum. (e) “Amam” signal autocorrelation time. (f) “Amam” signal probability distribution.

**Figure 4:**
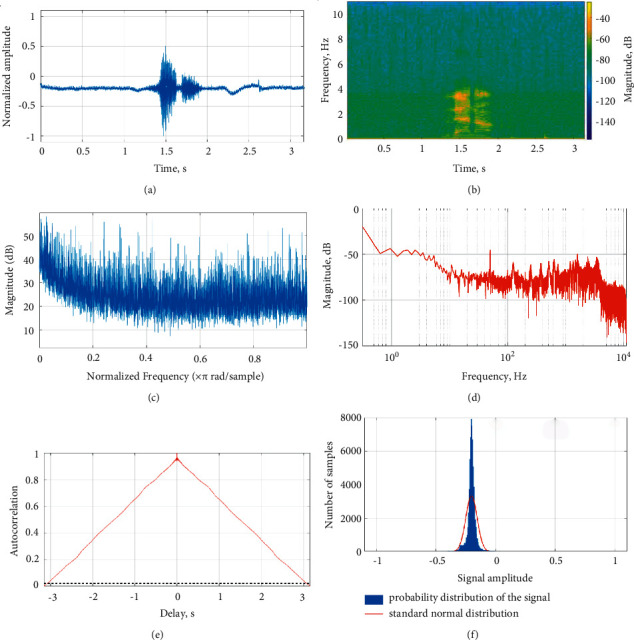
“Vena” speech signal waveform, spectrogram, magnitude response, amplitude spectrum, autocorrelation, and probability distribution in Table 2. (a) “Vena” speech signal. (b) “Vena” signal spectrogram. (c) “Vena” signal magnitude response. (d) “Vena” signal amplitude spectrum. (e) “Vena” signal autocorrelation time. (f) “Vena” signal probability distribution.

The Fourier domain analysis of BCM voice signals is from the right ramus, larynx, and right mastoid. The Fourier shows tone and phoneme variation of the speech signal. The low-frequency speech signal fails to conduct through bone compared to the high-frequency speech signal. The low-frequency speech signal and phoneme distort in the right ramus and right mastoid. However, the low- and high-frequency signals are conducted through the larynx to provide a clear representation of phoneme in speech signal. Each word of speech signal records for five times from different locations. The words were recorded at one-minute interval to reduce speaker fatigue. The recorded speech signal evaluates by the listener for speech intelligibility. The recorded speech signal and BCM signal were assessed with a slider scale. The listeners correlated recorded speech signal and BCM signal from the right ramus, right mastoid, and larynx with 72%, 84%, and 91% speech intelligibility. The right ramus, right mastoid, and larynx conducted speech signal showed mean speech intelligibility of 75%, 87%, and 92%, respectively. The BCM speech signal from the larynx shows higher speech intelligibility compared to other regions. The different bone locations are shown in Figure 2. The speech signal is acquired from the larynx bone train with CapusleNet, UNet, and S-Net for automatic speech recognition.  Figure 3(a) shows the acquired speech signal of “Amam” Tamil word. The speech signal spectrogram in Figure 3(b) shows the phoneme of “Amam” word signal. The low-frequency component of the signal shows similar speech intelligibility compared to the speech signal acquired through the microphone. The BCM further reduces the presence of noise in the speech signal and shows the feature of spoken words since BCM is in direct contact with the larynx bone. The magnitude response of the speech signal shows the variation in “Amam” word phoneme in the range of 10 to 55 dB as in Figure 3(c). The amplitude spectrum signal shows the Fourier representation of the speech signal as in Figure 3(d). The Fourier representation of speech signal shows the time signature of word phoneme. The autocorrelation and probability distribution of the signal is shown in Figures 3(e) and 3(f). Similarly, the second word “Vena” acquired speech signal is shown in Figure 4(a). The spectrogram of “Vena” signal in Figure 4(b) shows speech intelligibility at 4 Hz. The magnitude of the signal ranges from 50 to 10 dB due to the initial low phoneme variation, “Ve.” The amplitude spectrum of speech signal shows the speech intelligibility of word phoneme in the range of −50 to −120 dB. The autocorrelation and probability distribution of “Vena” speech signal is shown in Figures 4(e) and 4(f). Similarly, the Tamil word “Iruku” has speech intelligibility at 5 Hz, and its magnitude response changes in the range of 50 to 20 dB. The “Illa” word has speech intelligibility at 7 Hz and magnitude response in the range of 50 to 25 dB. The “Enna” word has speech intelligibility at 4.5 Hz and magnitude response in the range of 60 to 10 dB. Table 2 shows the speech signal parameter of different Tamil words use for analysis.

### 4.1. CapsuleNet

**Figure 5:**
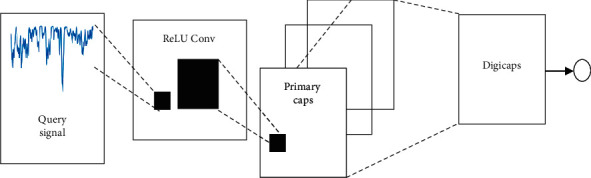
CapsuleNet architecture.

The CapsuleNet architecture is shown in Figure 5 which consists of the convolutional fully connected layer. The convolutional layers have 9 × 9 convolutional kernels and ReLU activation which extracts speech signal features from the BCM signal. The features are identified by feature detectors. The features apply as input to multidimensional lower order capsules. The lower order capsules followed by the primary capsule are made of 32-channel convolutional capsules. Every capsule consists of 8 convolutional units and produces a total of 256 × 81 convolutional units for speech recognition. The 6 × 6 capsules form 32 × 6 × 6 primary capsules and represent by equation (1). The output layer consists of 16D capsule which connects to all capsules in the layer. The parent capsules are zero initialised, and all capsules have zero probability for speech recognition. The learning loss and marginal loss of CapsuleNet minimize with Adam optimizer.

(1) $$\begin{array}{c} v_{j}=\frac{{\left\|s_{j}\right\|}^{2}}{1+{\left\|s_{j}\right\|}^{2}}\frac{s_{j}}{\left\|s_{j}\right\|} \end{array}$$

where *v*_*j*_ represents output produced by capsule *j* for input *S*_*j*_. The input *S*_*j*_ is weight adjusted by capsules to predict speech outcome. The prediction ${\hat{u}}_{j|i}$ form by the product of the *W*_*ij*_ weight matrix and output *u*_*i*_ is represented by the following equation:

(2) $$\begin{array}{c} s_{j}=\sum_{i}c_{ij}{\hat{u}}_{j/i},{\hat{u}}_{j/i}=W_{ij}u_{i}, \end{array}$$

where *c*_*ij*_ represents coupling coefficients represented by the following equation:

(3) $$\begin{array}{c} c_{ij}=\frac{\exp\left(b_{ij}\right)}{\sum_{k}\exp\left(b_{ik}\right)}, \end{array}$$

The coupling coefficient in capsules forms by routing softmax. The routing softmax has logits (*b*_*ij*_) and determines the capsule coupling among layers. The capsule determines the features in the input speech signal based on the instantiation vector. The margin loss (Lk) for multiple features in the input signal for each capsule *k* is represented by the following equation:

(4) $$\begin{array}{c} L_{k}=T_{k}\max{\left(0,m^{+}-\left\|v_{k}\right\|\right)}^{2}+\lambda \left(1-T_{k}\right)\max{\left(0,\left\|v_{k}\right\|-m^{-}\right)}^{2}. \end{array}$$

**Figure 6:**
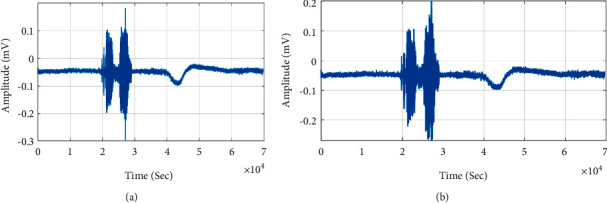
CapsuleNet Speech recognition. (a) “Amam” query speech signal. (b) Recognised speech signal.

Figures 6(a) and 6(b) show CapsuleNet retrieved Tamil word “Amam” for input query BCM signal. The CapsuleNet recognition of BCM from larynx bone has high accuracy compared to the BCM signal acquired from right ramus and mastoid bone. The signal from the larynx bone has average mean and crest factor of 15.30 dB and −0.17091 since the speech signal characteristics do not affect by background noise and bone conduction.

### 4.2. UNet

**Figure 7:**
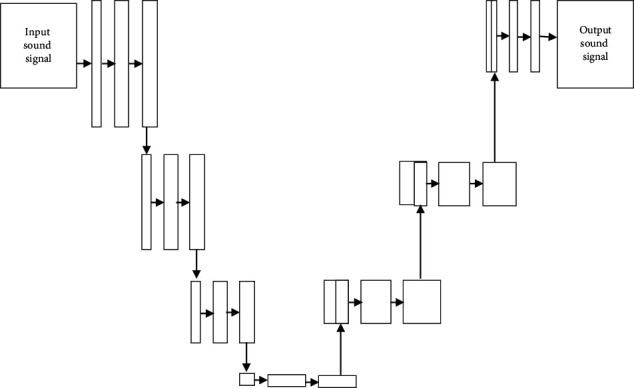
UNet architecture.

**Figure 8:**
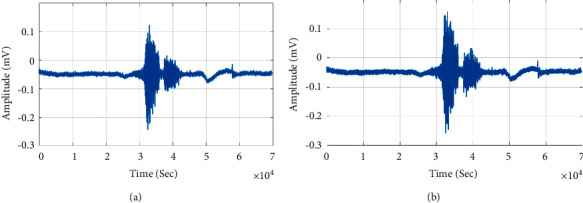
UNet speech recognition. (a) “Vena” query speech signal. (b) Recognised speech signal.

Figure 7 shows the architecture of UNet. The UNet forms by a convolutional neural network (CNN) in “U” shape. The UNet has paths namely contraction path (or) encoder and expansion path (or) decoder. The encoder performs activation, convolution, and pooling which captures the input BCM speech signal. The decoder extracts spectral features and spatial information to feature map of the speech signal by up convolution and concatenation process. The feature map has rich spectral information in the encoder phase, and the intermediate low-level features are combined in the decoder phase are combined to form feature channels. The feature samples propagate speech information to higher layers of CNN. The input speech signal is preprocessed to remove background noise and unsampled by a factor of two to form an enhanced feature map. The enhanced feature map formed from the encoder is concatenated. The concatenated feature map upsamples by two factors before applying to convolutional layers. The process continues till vocal spectral content is present for speech recognition. The UNet consists of a convolutional network with two 3 × 3 convolutions, pooling and rectified linear units to perform downsampling. The downsampling process increases feature channels, and upsampling at the decoder performs by 2 × 2 convolution which reduces redundant features of the speech signal. Figures 8(a) and 8(b) show query and recognised speech signal of the Tamil word “Vena.” The speech intelligibility of UNet is low for ramus and mastoid bone. The larynx bone has higher speech intelligibility with respect to a phoneme in the speech signal.

### 4.3. S-Net

**Figure 9:**
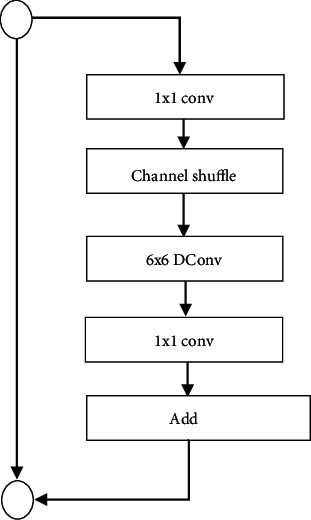
S-Net architecture.

**Figure 10:**
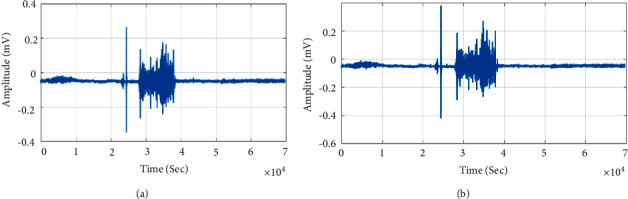
S-Net speech recognition. (a) Query speech signal. (b) Recognised speech signal.

S-Net works with Shufflenet for feature detection as shown in Figure 9. The S-Net provides efficient computing in dense convolutions (1 × 1). The S-Net and Shufflenet use pointwise group convolutions and channel shuffle operation for speech input weight adjustment in feature channels. The Shufflenet block consists of a 6 × 6 layer with 6 × 6 convolution to map speech input in the feature map. The Shufflenet performs average pooling and channel concatenation to handle the feature dimension of input speech. The Shufflenet has less complexity as it requires minimal FLOPs and convolutions. Figures 10(a) and 10(b) show speech recognition of S-Net for “Illai” Tamil word. The S-Net clearly recognises signals acquired from larynx bone compared to other bones.

### 4.4. Support Vector Machine (SVM)

The SVM is a supervised linear classifier. The SVM recognises features and patterns in signals based on supervised learning. The SVM separates dimensional data by hyperplane into a different class. The hyperplane separates nonlinear data by projecting data to higher dimensional space. The high-dimensional space forms by kernel-induced feature space. The kernels namely dot product, RBF, and polynomial kernel implement to classify nonlinear data. The dot product, RBF, and polynomial kernel represent by equations (5)–(7). The data projection into high-dimensional space causes overfitting. The overfitting overcomes by the dot product. The SVM performs well to classify unknown data and likelihood can be calculated.

*K*(*x*, *x*) = *x Æ x¢*;

(5) $$\begin{array}{c} K\left(x,x^{\prime }\right)=x.x^{\prime }, \end{array}$$

(6) $$\begin{array}{c} K\left(x,x^{\prime }\right)={\left(x.x^{\prime }+1\right)}^{d}, \end{array}$$

where *d* represents positive integer degree of kernel.

(7) $$\begin{array}{c} K\left(x,x^{\prime }\right)=\exp\left(\frac{-{\left|\left|x-x^{\prime }\right|\right|}^{2}}{\sigma^{2}}\right), \end{array}$$

where *σ* is a real number.

#### 4.4.1. Least Square Support Vector Machine (LSSVM)

The LSSVM is an improved version of SVM which uses the least square cost function. The LSSVM uses linear equations to train data instead of a quadratic equation as in SVM. The LSSVM with RBF kernel provides accurate prediction with minimal training time compared to SVM.

#### 4.4.2. Support Vector Regression (SVR)

SVR trains by symmetric loss function for prediction. The SVR uses the spare solution, kernels, and support vectors for function estimation. The SVR obtains from SVM by e-tube. E-tube is an e-insensitive region of the function. The e-tube reformulates to determine the best-valued function with minimal prediction error. The e-tube predicts function such that the tube has multiple training instances. The function represents by the following equation:

(8) $$\begin{array}{c} \underset{w}{\min}\frac{1}{2}{\left\|w\right\|}^{2}, \end{array}$$

where ‖*w*‖ represents the magnitude of the vector being approximated.  Table 3 shows voice and BCM signal correlation with LSSVM, SVM, and SVR.

## 5. Conclusion

The study describes the identification of optimal bone to provide speech intelligibility with BCM. The BCM speech signal was acquired from three different bone locations namely right ramus, larynx, and right mastoid. The BCM-conducted speech signal from different bones was rated for speech intelligibility by listeners, spectral analysis of the signal, and deep learning architectures namely CapsuleNet, UNet, and S-Net. The larynx bone-conducted speech signal showed a mean speech intelligibility of 92%. The CapsuleNet, UNet, and S-Net recognised Tamil word accurately for BCM signals obtained from larynx bone accurately compared to other ramus and mastoid. In the future, we will work to improve the model performance of this system and expand its application to more severe environments.

## Figures and Tables

**Table 1:** Related works.

Reference	Sensor	Problem	Method
[8]	Stethoscope and acoustic sensor	Lombard reflex on nonaudible murmur recognition in the presence of noise	Evaluation of nonaudible murmur microphone robustness with real and simulated noisy data
[9]	Softband bone conducted hearing device	Analyze auditory, speech development of bilateral microtia-affected children	The speech development of children assesses with a meaningful auditory integration scale and speech intelligibility rating
[10]	Throat, acoustic microphone	Improve throat acoustic microphone speech recognition	The throat and acoustic microphone correlate to extract acoustic feature vector for speech recognition
[11]	Baha attract bone hearing system	Speech recognition of wireless bluetooth device in patients using a baha attract bone hearing system and traditional hearing aid	Speech perception, recognition of Korean sentences were performed in quiet and noisy conditions
[12]	Bonebridge™ MED-EL	Speech recognition performance comparison of semiimplanted bonebridge MED-EL and adhesive bone-conduction device	Free-field audiometry test was conducted with speech, noise produced through loud speaker
[13]	Air and bone conduction microphone	Evaluate enhanced speech quality signal	The equalised bone conducted speech produced by maximum likelihood and bone conducted estimator for high and low SNR conditions, respectively. The equalised bone conducted speech quality evaluates with wiener gain and priori SNR estimator
[14]	Bone conducted microphone	Nonstationary noise suppression of speech signal	Supress noise in speech signal by selection of speech codebook based on noise free bone conducted microphone reference signal
[15]	Bone conducted microphone	Low frequency noise suppression	Supress low frequency noise namely colour, multitasker babble, and car from speech signal with bone conducted speech. The low noise frequency signal present in air-conducted speech is replaced with bone-conducted speech
Proposed	MEMS acoustic vibration transducer	Tamil word recognition	One syllable, two-syllable, and three-syllable Tamil speech recognize with CapsuleNet, UNet, and S-Net

**Table 2:** Signal parameters of different Tamil words.

Syllable	Sigma	Mu	Crest factor *Q* (dB)	Dynamic range (dB)	Autocorrelation time (sec)
Amam	0.055079	−0.16242	15.3147	79.7327	3.0891
Vena	0.047551	−0.20297	13.6193	72.0075	3.0784
Iruku	0.043221	−0.24713	12.0106	66.6918	3.0838
Illa	0.042194	−0.14086	16.6509	75.1304	3.0865
Enna	0.050101	−0.10114	18.9488	83.7131	3.0449

**Table 3:** Voice and BCM signal correlation with LSSVM, SVM, and SVR.

Correlation between voice and BCM signal				
Tamil words and syllabi	Architecture			Correlation algorithm (%)
	S-Net	UNet	CapsuleNet	
Amam (2 syllabi)	83.29	85.62	87.52	LSSVM
Vena (2 syllabi)	81.56	84.58	88.15	
Iruku (2 syllabi)	82.69	87.56	92.15	
Illa (2 syllabi)	83.59	87.91	93.45	
Enna (2 syllabi)	83.15	88.15	91.25	
Engae (2 syllabi)	82.18	88.94	93.58	
Naan (2 syllabi)	83.29	89.18	94.59	
Va (1 syllabi)	92.6	93.25	96.12	SVM
Engae va (3 syllabi)	91.2	92.89	97.25	SVR

## Data Availability

The datasets used and/or analyzed during the current study are available from the corresponding author on reasonable request.
